# Monthly Summary

**Published:** 1889-02

**Authors:** 


### Monthly Summary.

Mistaken Diagnosis.—*By Ed. L. Fairley, L. D. S.*—An interesting case of mistaken diagnosis by two surgeons in New York came under my notice recently. Miss E., aged 29, came to me about some carious teeth in the upper jaw of the right side. Noticing a large swelling on the left side of the same jaw, I inquired as to its history. The patient was more than usually intelligent on the subject. It had first attracted the attention of a surgeon a year ago, who declared it was an osseous tumor; then last July, of another who said it was a cystic tumor; both holding that a surgical operation for removal would be necessary. There were no decayed or undeveloped teeth on that side of the jaw; no functional disturbance; no congestion of the gums; no pain. About a year ago a slight pain had been felt in the first molar, but it was said to be entirely sympathetic. In the meantime the so-called "tumor" was slowly growing in size. The patient got so accustomed to its presence that I fancy she rather cherished it, and would not have thought any more for the present about it, had I not warned her of its possibilities.

Upon examination, I found imperfect calcification of the first molar, and the indications of a dead pulp, but very slight response to tapping. A cavity was drilled through the crown into the pulp cavity. The pulp was mummified and dry as punk. The canals of both roots were enlarged down to and through the apex; and by the use of peroxide of hydrogen and the usual treatment, I treated the tooth which was the cause of all the trouble. The swelling was reduced in a few weeks.—*Dominion Dental Journal.*

The Care of the Nails.—Very few people know how to properly care for the nails. In cleaning them, a sharp knife ought never to be employed, and between the ends of the nails and the fingers the space should be filled with soap, and then removed by brushing with the so-called nail-brush.

Many improperly cut away that part of the flesh which grows over the nail from the bottom; but it should be simply pressed backward, and sufficiently to show the white part, considered by some to be a mark of beauty. If the flesh is adherent to the nail the operation may be facilitated by passing the sharp point of a knife underneath the fold of flesh and separating it from its attachments. With this done, it can be pushed back more readily, scissors should never be used to cut the nails; that should be done only with a sharp penknife.—*Boston Journal of Health.*

---

HURRY IS THE CAUSE.—The evils of hurry are manifestly among the causes of poor and inefficient work. From a habit of hurry some people, having, or imagining they have, more to do in a given time than can be done properly, grow confused, agitated and nervous; and, under this pressure, they proceed with the work in hand without the requisite deliberation and care, perhaps omitting parts of it (sometimes important parts), and producing at least an imperfect and inferior result, which can neither be permanent nor satisfactory. It is not too much to say that a large proportion of the unhappiness, the ignorance, the loss of property, and even the loss of life that is endured in the world is to be traced to the hurry and drive which characterize so much of the labor performed.—*Press and Printer.*

---

FOOD-CRANKS.—The vegetarians are at it again, trying to persuade people to give up eating meat. Of all cranks on earth food cranks are the wretchedest. They pass their miserable lives in an insane effort to find out what agrees with them. As a rule nothing does. They remind one of the horse that was fed on saw-dust—just when he learned to eat it he died. They are afraid to eat a wholesome meal. Morbid dread of swallowing some impurity takes away all pleasure of living. Dyspepsia like a starving vulture sits upon their congested maws and pecks at their vitals. The sorriest specimens of humanity I have seen are those that patronize so-called health-food stores. How they survive is a mystery.—*N. Y. Tribune.*

WHO IS MY NEIGHBOR?—Human beings touch at numerous points. Their brotherhood may be denied or ignored, but it perpetually reasserts itself. It is a truth of physical science as well as of Divine revelation that unto himself no man can live or die. In the matter of communicable diseases, this matter assumes an awful significance. Thus, recently a case was reported of a child, a member of a large family, who was taken sick with scarlet fever on reaching a large city far from home. In vain the parents sought in that city for a place, public or private, in which to care for this sick child. There was no such place, and the father was compelled to take his child in a crowded sleeping-car on a long journey. One shudders to think of the exposure to numerous persons, old and young, to the contagion of this disease. The child was a neighbor to these and doubtless to some it transferred the deadly poison.

Carlyle tells the story of a poor widow thus: Her husband died of typhus, in one of the lanes of Edinburgh, and she wandered about the town with her three children, seeking help, and finding none, returned to her lane and died there. Her journeying started an epidemic that killed seventeen persons. Those to whom this widow appealed denied her sisterhood to them, but she proved her sisterhood, as her typhus fever killed them.

Numerous instances are fresh in every mind, proving the same truth. Cholera, yellow fever, small pox, etc., are each constantly proving to the world that each individual may be a neighbor to one distant thousands of miles. It is difficult to prove that any one individual in the world may not be a neighbor to any other person, by communicating to the same some diseased germ or poison. So completely has rapid transit annihilated time and space, that persons and articles carrying disease are met at most unlooked for places and under unwonted circumstances.

Admitting this, it is a question of personal import to every person that all other persons in the world be free from the contagium of disease. Hence, the one thing that should meet the heartiest support of all is the cleanliness of the person and abodes of the entire race. Unless this can be secured, commercial and social intercourse is fraught with danger.

What shall I do with my dirty neighbor? This is a question of the times, which all sanitarians are seeking to answer, and to which at another time we shall direct attention.—*American Lancet.*

---

EARLY RISING AND LONGEVITY.—Professor Humphry's recent Collective Investigation Report on Aged Persons, contains some very positive evidence on a matter which has already engaged the attention of moralists as well as physicians. "The opportunity for nutrition to do its restorative work was in nearly all provided by the faculty of 'good sleeping,' to which was commonly added its appropriate attendant, the habit of 'early rising.'" Thus there is a relation between early rising and longevity. No doubt many people will hastily seize upon the sentence just quoted, and employ it in edifying lectures or essays for the perusal of youth, or embody it in popular medical works. Important qualifications follow in Dr. Humphry's report, but they are likely to be overlooked. Doubtless the habit of early rising is, in itself, healthy; most of all, it is a good sign of health when it evidently signifies rapid recovery from fatigue. Again, it usually denotes a strong will, the gift as a rule of a good physical constitution, or at least the safeguard of average bodily strength. Late risers are generally either invalids or persons of bad habits, idlers who are never free from other vices besides idleness. The nervous exhaustion which keeps a man wakeful throughout the small hours produces sleep late in the morning. This exhaustion is invariably due to one of several life-shortening influences, especially anxiety or indiscretion in diet or drink. Early rising is thus rather one effect of certain favorable influences, another result of which is longevity, than a cause of longevity. To turn a weakly man out of bed every morning at seven o'clock will not prolong his life. It will be noted that by "good sleeping" Professor Humphry signifies quick sleeping, "that is, the reparative work which has to be done in sleep is done briskly and well." Here, again, we have an effect of a cause; but preventing a weakly subject from sleeping more than four or five hours nightly would not cause him to live long, but would rather tend to shorten his life. Equally important are Professor Humphry's observations which show that by "early" he does not entirely mean the time by the clock. The word "has a relative significance with reference to the time of going to bed. A person who retires to rest four hours after midnight and gets up at 10 a. m. may be strictly regarded as an 'early riser.'" Thus, early rising is synonymous in long life histories, with short sleeping, which means rapid recovery from fatigue, a sign of bodily strength. These scientific facts in no wise contradict the alleged value of early rising as a practice to be cultivated by all persons in good health. It is excellent as moral discipline, and eminently healthy as a matter of fact. Most persons will eat three meals a day. When a man gets up late those meals will probably follow each other at too short interval to be wholesome. When he is an early riser it will probably be otherwise. He can enjoy a good breakfast, and by the time for his lunch or mid-day dinner he will have an honest appetite again.—*British Medical Journal.*

---

REFLEX PAIN FOLLOWING THE EXTRACTION OF A TOOTH.—The following case came under my notice whilst a pupil with Dr. Parkinson, England. In July last John R.—age 19— serving the occupation of gardener, and undergoing treatment prior to filling, in the course of which I had the misfortune to leave in the posterior buccal root, a portion of a nerve broach, so small that at the time I was not aware of the accident. A few days later the tooth was removed, when nothing unusual occurred to excite attention. The next day the patient returned, complaining of pain in the vacated tooth socket, and also of pain in the testicles; the latter he felt to a very slight extent when the tooth was extracted. He was told to come on the following day but failed to do so, and shortly afterwards went to the sea side. He returned from Hastings a month later, better, and called at the surgery, when I learned that the pain in the two situations continued for a month after the tooth was removed, and generally appeared at the two places simultaneously. There were no physical alterations in the glands; he had gonorrhœa ten years ago, from which he duly recovered and is now married.—*V. A. Latham.*

---

MEDICAL MEN AND DENTISTS—The question has been recently raised as to the scale of fees to be charged by medical men to dentists and their families. We do not remember to have seen the question formally put before, but now that this has been done we do not hesitate to express an opinion that dentists ought to be considered more or less in the light of brother practitioners. Their conduct towards medical men who are in need of their services is almost invariably characterized by courtesy and consideration, and the opportunity to reciprocate is one which should be seized with *empressement.* The status of dentists has made vast progress during the past few years, and their social position is now more in accordance with the highly-skilled services which they render to a suffering public. Dentistry is, properly speaking, but a department of surgery, and by no means the least useful. Dentists do more for our comfort and health than do many more purely surgical operations, and they do it moreover with an ease and a certainty which the others have yet to attain.—*Medical Press.*

---

MISSIONARY DENTISTS.—The favorable reception of missionaries of the Gospel among heathen nations has of late years been greatly promoted by sending as heralds regular graduates of medicine, who, by the exercise of their skill in the healing art, have won the confidence and esteem of most barbarous and blood thirsty tribes. The practice of dentistry is likewise proving a means for peacefully introducing the missionary into the good graces of suffering savages, and in a recent tour of the island of Formosa it was a not infrequent preliminary to religious services for the missionary to extract fifty or more teeth. The radical relief of toothache, by simple and obvious means, may well excite heartfelt gratitude in the sufferer, and engender such confidence in the friendly disposition of the dentist that easy access will be gained for the faithful ministration of the Gospel teacher. The attention of the various missionary boards might be profitably directed to the obvious advantages of having more of their messengers duly qualified and equipped for the gratuitous practice of dental surgery among the heathen.—H.

